# Ulocuplumab (BMS-936564 / MDX1338): a fully human anti-CXCR4 antibody induces cell death in chronic lymphocytic leukemia mediated through a reactive oxygen species-dependent pathway

**DOI:** 10.18632/oncotarget.6465

**Published:** 2015-12-04

**Authors:** Manoj K. Kashyap, Deepak Kumar, Harrison Jones, Carlos I. Amaya-Chanaga, Michael Y. Choi, Johanna Melo-Cardenas, Amine Ale-Ali, Michelle R. Kuhne, Peter Sabbatini, Lewis J. Cohen, Suresh G. Shelat, Laura Z. Rassenti, Thomas J. Kipps, Pina M. Cardarelli, Januario E. Castro

**Affiliations:** ^1^ UCSD-Moores Cancer Center, La Jolla, CA, USA; ^2^ CLL Research Consortium, La Jolla, CA, USA; ^3^ Bristol-Myers Squibb, Department of Cell Biology and Physiology, Redwood City, CA, USA; ^4^ Department of Early Clinical and Translational Research, Bristol-Myers Squibb, Princeton, NJ, USA

**Keywords:** Ulocuplumab, BMS-936564, reactive oxygen species, chronic lymphocytic leukemia, CXCR4

## Abstract

The CXCR4 receptor (Chemokine C-X-C motif receptor 4) is highly expressed in different hematological malignancies including chronic lymphocytic leukemia (CLL). The CXCR4 ligand (CXCL12) stimulates CXCR4 promoting cell survival and proliferation, and may contribute to the tropism of leukemia cells towards lymphoid tissues. Therefore, strategies targeting CXCR4 may constitute an effective therapeutic approach for CLL. To address that question, we studied the effect of Ulocuplumab (BMS-936564), a fully human IgG4 anti-CXCR4 antibody, using a stroma – CLL cells co-culture model. We found that Ulocuplumab (BMS-936564) inhibited CXCL12 mediated CXCR4 activation-migration of CLL cells at nanomolar concentrations. This effect was comparable to AMD3100 (Plerixafor - Mozobil), a small molecule CXCR4 inhibitor. However, Ulocuplumab (BMS-936564) but not AMD3100 induced apoptosis in CLL at nanomolar concentrations in the presence or absence of stromal cell support. This pro-apoptotic effect was independent of CLL high-risk prognostic markers, was associated with production of reactive oxygen species and did not require caspase activation. Overall, these findings are evidence that Ulocuplumab (BMS-936564) has biological activity in CLL, highlight the relevance of the CXCR4-CXCL12 pathway as a therapeutic target in CLL, and provide biological rationale for ongoing clinical trials in CLL and other hematological malignancies.

## INTRODUCTION

Chronic lymphocytic leukemia (CLL) is the most frequent adult leukemia and is characterized by accumulation of aberrant B-lymphocytes [1]. Stromal cell support of CLL cells has shown survival through membrane-associated factors such as CXCR4 (chemokine C-X-C motif receptor 4). CXCR4 is a G protein coupled receptor consisting of 7 transmembrane domains, [2] that is expressed in different cell types, including B cells, monocytes, T cells, neutrophils, macrophages, natural killer (NK) cells, endothelial, epithelial, CD34^+^ hematopoietic stem cells, and dendritic cells [3-6]. CXCR4 not only is expressed in CLL but also in a variety of cancers including acute myeloid leukemia, myeloma, lymphomas, clear cell renal cell carcinoma, breast, lung, colon, pancreatic, and ovarian cancer [7]. CXCR4 has a single ligand, CXCL12 (chemokine C-X-C motif ligand 12), [8] which is a homeostatic chemokine also known as stromal cell-derived factor 1 (SDF-1). CXCL12 regulates hematopoietic cell trafficking, secondary lymphoid tissue architecture, and homing of hematopoietic stem cells (HSC) to the bone marrow [5]. In addition, CXCL12 mediates survival, proliferation of B-cell progenitors and CLL cells, [9, 10] and participates *in vitro* in stromal cell dependent resistance to cytotoxic drugs like fludarabine (F-ara-A), [6] or steroids [11]. Therefore, CXCL12 mediated activation of CXCR4 may favor resistance to therapy in CLL patients by promoting and maintaining minimal residual disease [12-14].

Several anti-CXCR4 antibodies are currently available including MAbs 6H7, 7D4, 1D9, and 12G5, [15-16] which are used primarily as reagents for flow cytometry or immunohistochemistry. Ulocuplumab (BMS-936564, Bristol-Myers Squibb) is a novel IgG4 fully human monoclonal antibody that binds to the second extracellular loop of CXCR4.

Ulocuplumab (BMS-936564) binds to CXCR4 at low nanomolar concentrations compared to other commercially available antibodies (*e.g.* 1D9). This antibody prevents the binding of CXCL12 and inhibits calcium flux mediated cell motility and migration [17]. The Ulocuplumab (BMS-936564) antibody is an IgG4 [17], that lacks complement-dependent cytotoxicity activity (CDC) and antibody-dependent cell-mediated cytotoxicity (ADCC) activity as confirmed in the current study in primary CLL and Ramos cell lines. Therefore, most of its anti-cancer activity is possibly mediated by direct binding to CXCR4 and interference with the interaction to its ligand (CXCL12). Here, we present our studies with primary leukemia cells from CLL patients using Ulocuplumab (BMS-936564) in culture conditions that resemble the leukemia microenvironment.

## RESULTS

### Expression of CXCR4 and CXCL12 in CLL, normal B, and stroma-NK-tert cells

**Figure 1:**
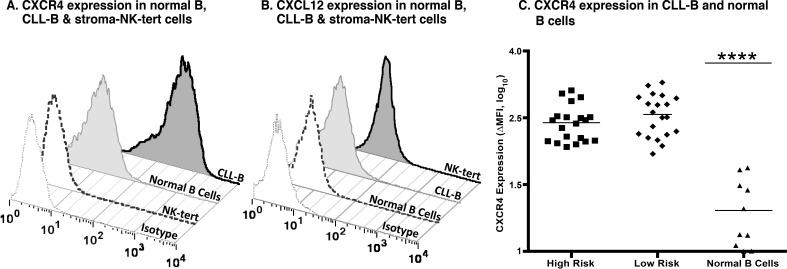
CXCR4 and CXCL12 expression in CLL, normal B and stroma cells **A.** Expression of CXCR4 after surface staining using an anti-CXCR4 antibody in B cells derived from CLL patients, healthy volunteers, and stroma-NK-tert cells, as compared to the respective isotype. **B.** Expression of CXCL12 after intracellular staining using an anti-CXCL12 antibody in B cells derived from CLL patients, and normal PBMCs from healthy volunteers, and stroma-NK-tert cells, as compared to the isotype. **C.** Panel shows the CXCR4 expression in samples from CLL patients with high risk and low risk characteristics and normal B cells. The line indicates the mean of each group.

Expression of CXCR4 and CXCL12 was assessed by flow cytometry in primary leukemia cells from patients with CLL as well as in normal B, and stroma-NK-tert cells (Figure 1A and 1B). Additionally, established cell lines used in our experiments as controls were evaluated for CXCR4 expression (Figure 1 and Supplementary Figure 1).

**Figure 2:**
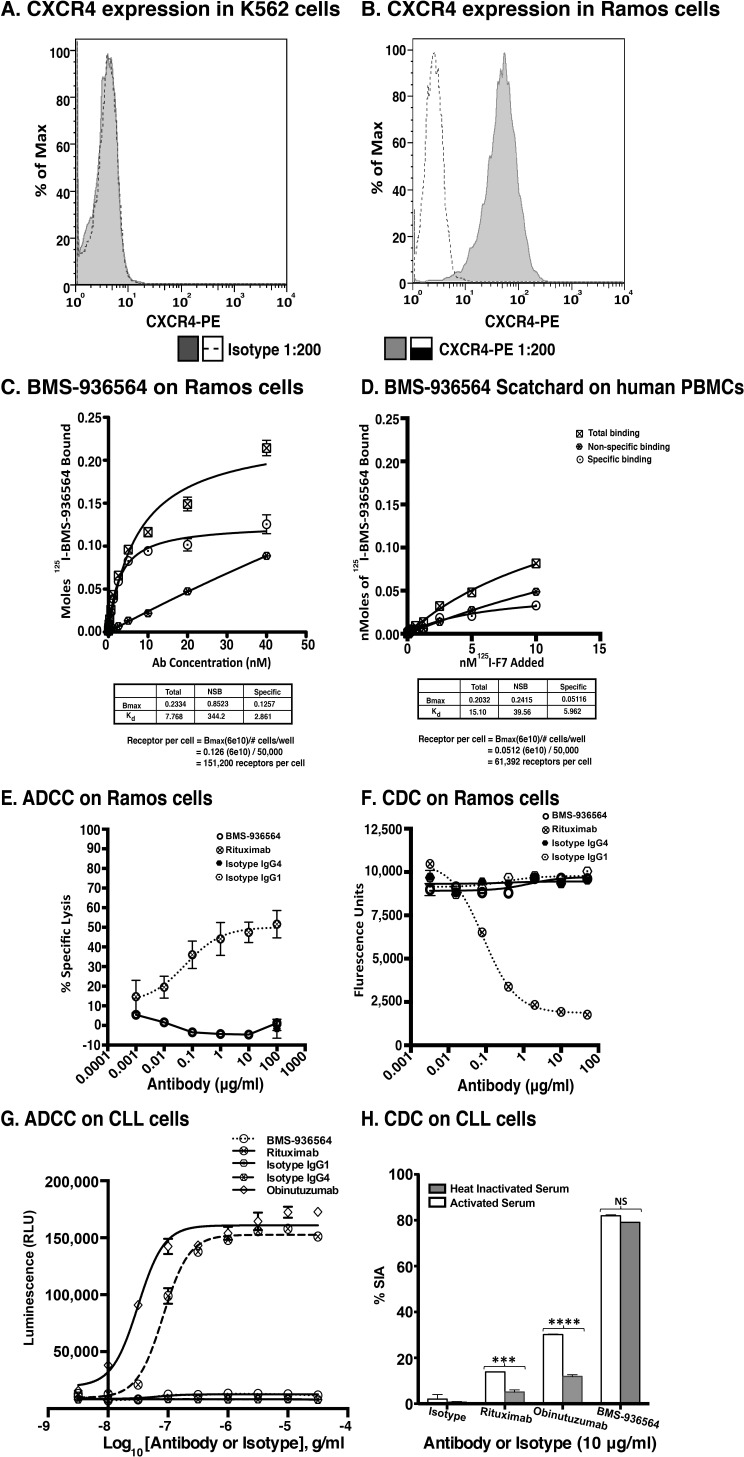
Scatchard analysis of Ulocuplumab (BMS-936564) binding to Ramos cells, human PBMCs and ADCC & CDC activity in Ramos cell line (Burkitt's lymphoma) **A.** CXCR4 Expression profiling was done using an anti-CXCR4 antibody for surface staining in K562 and **B.** Ramos cell lines followed by analysis of samples using flow cytometry. The CXCR4 expression is presented in form of ΔMFI. **C.** The affinity of Ulocuplumab (BMS-936564) was measured using endogenously expressed CXCR4 positive Ramos cells. The binding capacity of ^125^I-Ulocuplumab (BMS-936564) to CXCR4^+^ cells in presence or absence of excess unlabeled antibody was determined and a K_d_ of 2.9 nM and approximately 1.5×10^5^ receptors/cell was calculated. **D.** Normal peripheral blood cells were incubated with increasing concentrations of ^125^I-Ulocuplumab (BMS-936564). The counts per minute bound (CPM) in the presence or absence of unlabeled antibody was measured. Scatchard analysis of saturation binding curves was performed with Prism 4 GraphPad software (GraphPad software, San Diego, CA) using nonlinear regression analysis. The symbol 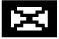, 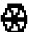 and 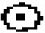 represent total binding (TB), non-saturable binding (NSB), and saturable binding (SB), respectively. **E.** Ramos cells (target) were labeled with bis(acetoxymethyl) 2,2′:6′,2″-terpyridine-6,6″-dicarboxylate (BADTA). Freshly isolated human peripheral blood mononuclear cells PBMCs (effector) were used for allotyping of the Ulocuplumab (BMS-936564). Human PBMCs were cultured with labeled Ramos cells at 1:50 ratio of target/effector cell (T/E) ratios in the presence or absence of rituximab, Ulocuplumab, or isotype (BMS-936564) for 1 h at 37°C. Ramos cells alone served as spontaneous release (SR) and Ramos cells lysed with 1% Triton X-100 served as total release (TR). The lysis was measured by using europium (Eu)-based detection. BMS-936564, Rituximab and their respective isotypes with varied concentrations were tested using Ramos cell line. **F.** Cell-based CDC assay of Ulocuplumab (BMS-936564) /Rituximab and their respective isotypes as controls: lysis of Ramos cells in the presence of human complement was measured by using Alamar Blue release. **G.** The stable transfected Jurkat cell line expressing FcgRIIIa and NFAT-RE luc was used as effector **E.** in an ADCC Reporter Bioassay from Promega. CLL cells (target, T) were plated in ratio of 1:1 with the effector cells. The effector: target cells were incubated in the presence or absence of rituximab or Ulocuplumab (BMS-936564), or obinutuzumab between 0.001-30 ug/ml of concentrations for 6 hrs at 37°C. Rituximab and obinutuzumab were used as positive controls. The reaction was developed by incubating the cells with Bio-Glo^TM^ reagent for 30 minutes at room temperature in dark. The plates were read on luminometer and following the background subtraction, relative-light units (RLU) were calculated for different antibodies/isotypes using GraphPad Prism software. **H.** Complement dependent cytotoxicity (CDC) for 10 μg/ml of either Ulocuplumab (BMS-936564), rituximab, obinutuzumab or isotype was tested in CLL cells after incubation with either 5% fresh human or heat inactivated serum (to denature complement) was measured by using CD19/CD5/Annexin V staining followed by flow cytomerty analysis. Rituximab and obinutuzumab were used as positive controls. The data are the mean and SD of triplicate cultures. The statistical data was analyzed using Bonferroni correction test in GraphPad Prism software.

We observed that the level of expression of CXCR4 was higher in CLL by at least 8 fold when compared to normal B cells. As expected, CXCL12 expression was not detected in CLL cells but was high in stroma-NK-tert cells (Figure 1B), and other leukemia and lymphoma cell lines (Figure 2A-2B and Supplementary Figure 2).

We evaluated a group of 20 patients categorized as CLL-HR and 20 patients categorized as CLL-LR (defined by using prognostic markers discussed above). We observed that the level of CXCR4 expression was independent of prognostic factors with an average ΔMFI (mean fluorescence intensity) of 432.2 (95% CI 314.5-549.9) (Figure 1C). There was no significant difference between CLL-HR and CLL-LR subtypes of CLL, but there was a significant difference between CLL subtypes versus normal B cells (*p* < 0.0001) with a level of expression that was 8 fold lower compared with CLL samples (ΔMFI average of 26.07 - 95% CI 11.6-40.5, *p* <0.01).

**Table 1:** List of cell lines used in the study

Name of Cell Line	Leukemia/Lymphoma	Cell Type	Media for Growth
MEC1	CLL	Lymphoblast	RPMI with 10% FBS and 1% Pen-Strep
K562	CML	Lymphoblast	
JVM2	MCL	Lymphoblast	
Raji	BL	Lymphoblast-like cells	
Ramos	BL	B lymphocytes	
Jurkat	Acute T cell leukemia	T lymphocyte	
Granta	BCL	B cell lymphoma	
EW36	BCL	B cell lymphoma	
Daudi	BL	B lymphoblast	
Mino	MCL	Lymphoblast	
Namalwa	BCL	B cell lymphoma	
JeKo-1	MCL	Lymphoblast	

BL: Burkitt's lymphoma, BCL: B cell lymphoma, CLL: chronic lymphocytic leukemia, CML: chronic myelogenous leukemia, MCC: mantle cell lymphoma, FBS: fetal bovine serum

### Affinity and saturation binding of ^125^I-BMS-936564 to CXCR4

We determined affinity and saturation binding of Ulocuplumab (BMS-936564) to CXCR4 using a radiolabeled antibody ^125^I-BMS-936564 in Ramos cell line (Burkitt's lymphoma). In comparison to K562 (chronic myelogenous leukemia, Figure 2A), there was high level of CXCR4 expression in Ramos cell line (Figure 2B). Competitive affinity binding of Ulocuplumab (BMS-936564) showed a mean KD (the equilibrium dissociation) of 2.8 nM and approximately 150,000 CXCR4 receptors per cell in B-lymphoma cells (Ramos, Figure 2C). Specific binding affinity of this antibody to human PBMCs was lower - 5.9 nM (Figure 2D), and the number of receptors per cell that was approximately one third of that found in human PBMCs (61,000 per cell).

### Ulocuplumab (BMS-936564) (IgG4 antibody) lacks ADCC or CDC activity but induces apoptosis mediated by CXCR4 binding

Ulocuplumab (BMS-936564) was engineered as a fully human IgG4 antibody with the purpose to lack ADCC or CDC activity and to work as a *“*blocking antibody*”*. We tested this antibody *in vitro* to demonstrate the lack of those two important functions.

Ramos and PBMC effector cells were incubated with increasing concentrations of Ulocuplumab (BMS-936564) to evaluate antibody dependent cellular cytotoxicity (ADCC) (Figure 2E), or with complement (CDC) (Figure 2F). Ulocuplumab (BMS-936564) did not induce cytotoxicity in the CLL-B cells (target cells) mediated by effector cell mechanisms (ADCC, Figure 2G) or by complement (CDC, Figure 2H).

**Figure 3:**
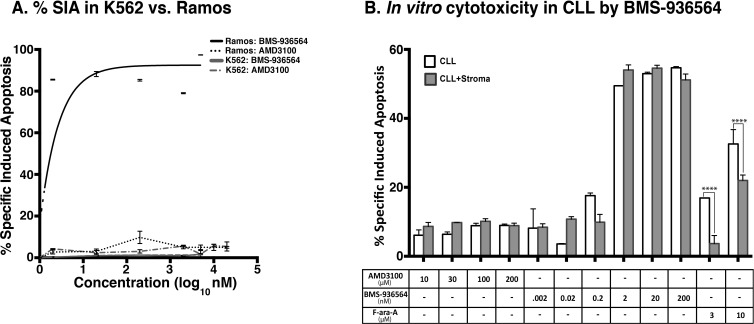
% SIA induction by Ulocuplumab (BMS-936564) is CXCR4 specific as observed in Ramos and primary CLL cells but not in K562 **A.** Ramos and K562 cell lines were treated with different concentrations of Ulocuplumab (BMS-936564) and AMD3100 for 48 hrs followed by running the samples on flow cytometer to study the % SIA. Treatment with AMD3100 in Ramos/K562/CLL did not induce significant % SIA as compared to Ulocuplumab (BMS-936564). In contrast, Ulocuplumab (BMS-936564) induced significant % SIA in Ramos and CLL cells (CXCR4+), but not in K562 (CXCR4-) as evaluated by flow cytometry). **B.** The CLL cells cultured either alone or with stromal cell support were treated with Ulocuplumab (BMS-936564) or AMD3100 for 48 hrs at 37°C followed by flow cytometer for measurement of % SIA. AMD3100 does not induce significant % SIA in CLL as compared to Ulocuplumab (BMS-936564). Ulocuplumab (BMS-936564) induced significant amount of % SIA in CLL cells alone or CLL co-cultured with stromal cell support.

We analyzed whether direct binding of CXCR4 could trigger apoptosis. K562 (CXCR4^−^, Figure 2A), and Ramos (CXCR4^+^, Figure 2B) were incubated with increasing concentrations of Ulocuplumab (BMS-936564) and then analyzed for development of apoptosis using flow cytometry. Only Ramos cells, which express high levels of CXCR4, underwent apoptosis (IC_50_ 1.9 nM) while K562 cells did not show evidence of cell death even when the antibody was used at high mM concentrations (Figure 3A). Before screening the primary CLL cells, we carried out washout experiments using the equivalent *in vivo* achievable concentration of Ulocuplumab (BMS-936564) (67 nM or 10 ug/ml). We found that the cells started to show *in vitro* cytotoxic effect within 5-10 hrs of treatment. It seems that Ulocuplumab (BMS-936564) saturates the cells in terms of cell death as the changes in CLL or CLL co-cultured with stroma-NK-tert cells become steady (Supplementary Figure 3). Similar dose dependent pro-apoptotic activity was observed in primary leukemia cells from CLL patients (IC_50_ of 12.43 nM). Interestingly, the activity of Ulocuplumab (BMS-936564) was present whether or not the leukemia cells were cultured alone or with stromal cell support. This indicates that the activity of Ulocuplumab (BMS-936564) is likely due to direct binding to CXCR4 rather than interference of the CXCR4-CXCL12 receptor / ligand interaction. Moreover, this antibody was able to overcome fludarabine resistance conferred by stromal cells in this *in vitro* model (Figure 3B). AMD3100, which binds and inhibits signaling through CXCR4, was used as a control. Importantly, this molecule did not induce cell death in CLL or any of the cell lines tested (Figure 3B, and data not shown).

The pro-apoptotic activity of Ulocuplumab (BMS-936564) was stronger on different cell lines and CLL cells compared to other commercially available CXCR4 antibodies – 1D9,[18, 19] and 12G5 [20, 21]. In the case of 1D9 the IC_50_ was not achievable and for 12G5 was 32.1 nM in CLL cells (Supplementary Figure 4). Additionally, in screening of different leukemia/lymphoma cell lines, we found that Ulocuplumab (BMS-936564), as compared to 12G5 and 1D9 antibodies was, more effectively inducing *in vitro* cytotoxicity (Table 2).

**Table 2:** IC_50_ of Anti-CXCR4 antibodies (1D9, 12G5, and BMS-936564) in leukemia/lymphoma cell lines

Cell Lines	CXCR4 (ΔMFI)	IC_50_ (nM)		
		BMS-936564	12G5	1D9
JVM2	0.65	NA	NA	NA
MEC1	0.8	NA	NA	NA
K562	1.2	NA	NA	NA
Granta	4.88	25.39	19.3	45.96
JeKo-1	22.75	1.86	49.41	584.47
EW36	27.76	0.00348	1550	990
Daudi	29.78	0.000955	9.81	874.67
Mino	56.71	628.93	2650	3010
Jurkat	71.06	15.49	626.93	270.27
Ramos	76.43	3.01	24.91	1980
Raji	82.72	8.85	245.93	32.99
Namalwa	181.2	2.57	28.52	597

NA: IC_50_ not achievable

### Ulocuplumab (BMS-936564) preferentially induces apoptosis in CLL/cancer cell lines, but not in normal lymphocytes

**Figure 4:**
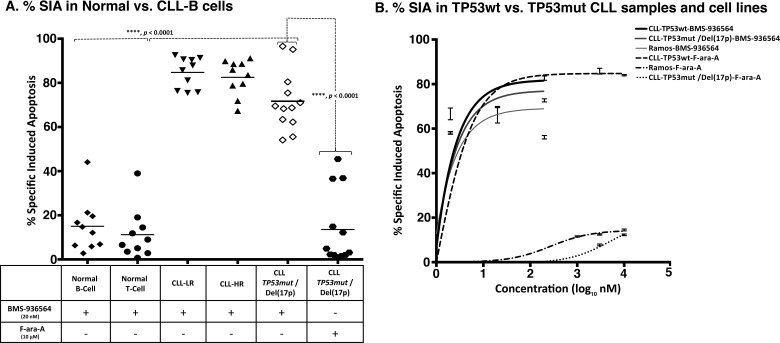
Ulocuplumab (BMS-936564) antibody induces apoptosis in CLL cells independent of TP53 status **A.** The primary leukemia B-cells were obtained from CLL patients with or without TP53 mutation or Del(17p) [*TP53mut* /Del(17p)]. Normal B and T lymphocytes were obtained from healthy donors. The % SIA was measured in normal B, T cells, CLL cells, *TP53mut* /Del(17p) CLL cells, alone or co-cultured with stroma-NK-tert cells after treatment with 200 nM of Ulocuplumab (BMS-936564) for 48 hrs of incubation at 37°C. % SIA of Normal B and T cells was significantly less when compared to CLL-LR, CLL-HR and *TP53mut* /Del(17p) patients, showing that Ulocuplumab (BMS-936564) has a good therapeutic index. Ulocuplumab (BMS-936564) induced apoptosis in CLL cells regardless of *TP53mut* /Del(17p) mutation status, while normal B or T cells showed statistically-lower level of apoptosis. The data shows the results of samples analyzed in duplicate with the mean and its respective standard deviations. **B.** Representative CLL samples from each subgroup - *TP53wt* or *TP53mut* /Del(17p) and Ramos *TP53* mutant were incubated for 48 hrs with the Ulocuplumab (BMS-936564) and Fludarabine (F-ara-A). Apoptosis was measured by flow cytometry.

Leukemia cells from CLL patients with CLL-HR or CLL-LR disease were incubated with Ulocuplumab (BMS-936564) and apoptosis was evaluated after 48 hrs of culture. Despite of the presence of high-risk prognostic factors [unmutated IgVH genes, high levels of ZAP-70 or *TP53mut* /Del(17p)], which are typically associated with poor clinical outcome and disease progression, we observed similar levels of apoptosis in CLL-HR and CLL-LR samples (Figure 4A). Interestingly, samples from patients with *TP53mut* /Del(17p) were sensitive to Ulocuplumab (BMS-936564), and showed comparable levels of apoptosis with CLL-HR and CLL-LR samples and the apoptosis levels were significantly higher than those observed after treatment with F-ara-A.

The IC_50_ for different CLL subgroups including low-risk, high-risk and *TP53mut* /Del(17p) were 4.9 nM, 9.8 nM, and 11.6 nM, respectively. In contrast, the IC_50_ was not achieved in normal B and T lymphocytes. On the other hand, normal B and T lymphocytes appear to be resistant to the cytotoxic activity of Ulocuplumab (BMS-936564) with apoptosis levels that were significantly lower than those observed in CLL samples. (Figure 4A).

### Ulocuplumab (BMS-936564) induced apoptosis is independent of p53 status

To show that the activity of Ulocuplumab (BMS-936564) was p53 independent, we incubated leukemia cells derived from either *TP53wt* or *TP53mut* /Del(17p) CLL patients and Ramos (*TP53mut*), and compared the level of apoptosis induced by F-ara-A, which is known to be a p53 dependent chemotherapy agent. [22] Ulocuplumab (BMS-936564) induced cell death in CLL *TP53wt*, *TP53mut* /Del(17p) and Ramos (*TP53mut*) with similar IC_50_ (2.7 nM, 2.7 nM, and 3 nM respectively). As expected, F-ara-A was active only in CLL *TP53wt* and the IC_50_ for F-ara-A in *TP53mut* /Del(17p) and Ramos (*TP53mut*) was not achieved even after using this compound at supra-physiological concentration (Figure 4B).

### Ulocuplumab (BMS-936564) inhibits F-actin polymerization and cell migration

The functional activity of Ulocuplumab (BMS-936564) was studied in CLL samples that underwent CXCR4 activation mediated by CXCL12. Using this model, CLL cells treated with CXCL12 underwent cytoskeletal reorganization measured by F-actin polymerization assay and chemotactic changes that were measured using a transwell migration assay.

**Figure 5:**
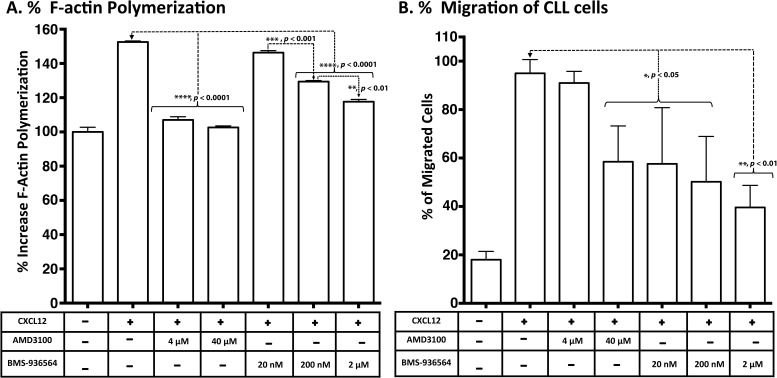
Inhibition of CXCL12-induced response and migration of primary CLL cells in a transwell assay by Ulocuplumab (BMS-936564) and AMD3100 **A**. Intracellular F-Actin was measured using FITC-labeled phalloidin in CD19/CD5- pre-labeled CLL cells after stimulation with CXCL12 (90 nM) for 15 seconds. CLL cells from 10 patients (5 HR & 5 LR) were treated with no compounds (control), AMD3100 (4-40 μM) or Ulocuplumab (BMS-936564) (20-2000 nM) prior to stimulation and the representative example of F-actin polymerization is shown. All samples are plotted relative to the mean fluorescence of the sample without any addition of the chemokine CXCL12. **B.** A total of 5×10^5^ cells per well were plated overnight before the experiment. The cells were incubated with Ulocuplumab (BMS-936564) and AMD3100 (using indicated concentrations) for one hour and loaded on to the transwell chamber and incubated for two hrs in the presence of CXCL12 (12.5 nM) or media control. After that, cells that migrated to the lower chamber were counted using flow cytometry.

CXCL12 induced an average increase in actin polymerization of 152.54% after stimulation with 90 nM CXCL12 for 15 seconds at 37°C. Ulocuplumab (BMS- 936564) at 200 nM (*p* < 0.0001) and 2 μM (*p* < 0.0001) concentrations significantly inhibited actin polymerization and reduced the peak response to CXCL12 by an average of 20-40%. Similarly, significant levels (*p* <0.0001) of inhibition were observed with AMD3100 at 4 μM and 40 μM concentrations), which was used as control for CXCR4 inhibition (Figure 5A).

CXCL12 induced chemotaxis and transwell migration in CLL cells with an average increase > 90% over base line (Figure 5B). Ulocuplumab (BMS-936564) (20 nM-2 μM) significantly inhibited cell migration by 40- 58%. AMD3100 (40 μM) also inhibited significantly (*p* <0.05) the migration compared to the peak stimulation after CXCL12 (Figure 5B).

### Ulocuplumab (BMS-936564) induction of programmed cell death (PCD) is caspase independent

**Figure 6:**
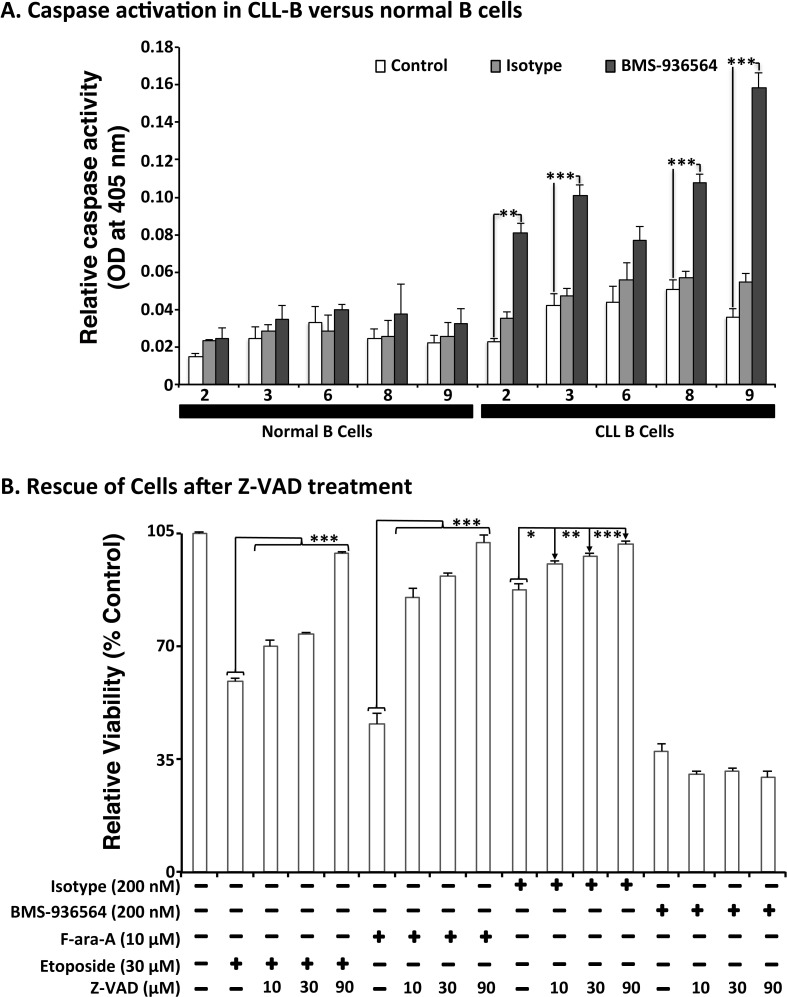
Effect of Ulocuplumab (BMS-936564)-mediated apoptosis in CLL cells is caspase-independent **A.** Cells were treated for 6 hrs with either 200 nM of Ulocuplumab (BMS-936564) or isotype control (IC). 100 μg of protein was taken from each treatment for caspase analysis. Caspase 2, 3, 8 and 9 were activated upon treatment with the Ulocuplumab (BMS-936564) antibody. There was a significant difference between normal versus CLL cells for caspase 2, 3, 8, and 9. Caspase activity of cells incubated in media only was used as a baseline control. Each value is expressed as mean ± S.D. of two independent experiments. **B.** CLL cells were incubated with Ulocuplumab (BMS-936564) (200 nM), F-ara-A (10 μM), and Etoposide (30 μM) for 48 hrs either alone or in combination with different concentrations of a pan-caspase inhibitor, Z-VAD-FMK (10, 30, 90 μM). CLL cells viability was analyzed by CD19^+^/CD5^+^/Annexin-V staining followed by flow cytometry. Statistical significance was determined by using Dunnett's multiple comparison test (* *p* < 0.05; ** *p*<0.01, *** *p* < 0.001). Z-VAD inhibited apoptosis in a dose dependent manner when apoptosis was induced by chemotherapy controls but not by Ulocuplumab (BMS-936564) (untreated media control vs. 0-90 μM Z-VAD, *p* < 0.0001).

To study the mechanism(s) of Ulocuplumab (BMS-936564) induced apoptosis, we evaluated caspase activation in CLL cells as well as in normal B cells after incubation with this antibody. Ulocuplumab (BMS-936564) induced significant caspase activation (caspase 2, 3, 8, 9) in CLL cells but not in normal B cells (Figure 6A). However, inhibition of caspase activation with Z-VAD did not block Ulocuplumab (BMS-936564) induced apoptosis suggesting that caspase activation is not required for the pro-apoptotic activity of this antibody (Figure 6B).

### Ulocuplumab (BMS-936564) induces cell death via production of reactive oxygen species (ROS) in CLL cells

**Figure 7:**
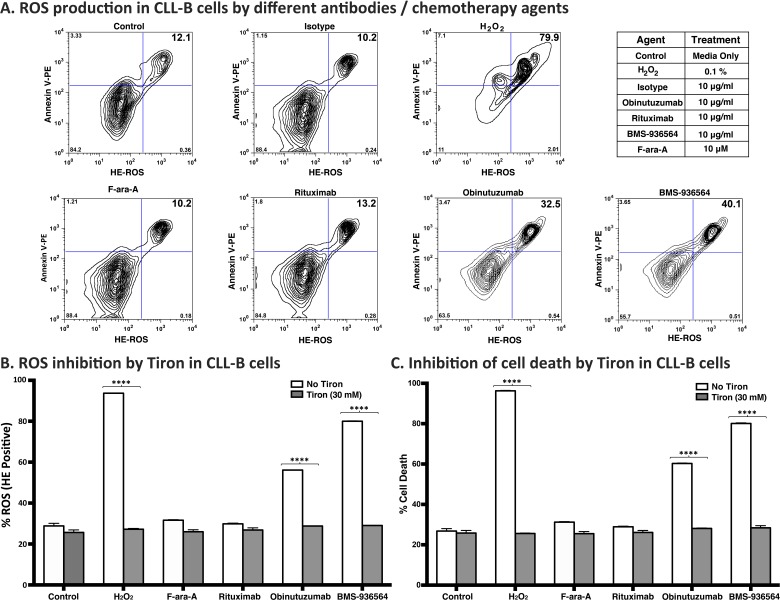
Reactive oxygen species (ROS) mediated mechanism of cell death by BMS-936564 in patients' derived CLL cells **A.** Primary CLL cells derived from patients were treated for 4 hrs either with 0.1% H_2_O_2_, 10 ug/ml of obinutuzumab, Rituximab, Ulocuplumab (BMS-936564) antibody or F-ara-A at the indicated concentrations. ROS production and cell death were assessed by flow cytometry. ROS^+^/Annexin-V^+^ double positive cells indicate the population of cell undergoing cell death with concurrent release of ROS (Upper right quadrant). Cells incubated in media only serve as base line control. This experiment represents typical data from three separate experiments conducted with duplicates per each incubation condition. **B.** The cells were incubated alone with Tiron (30 mM) or 10 μg/ml of either rituximab, obinutuzumab, Ulocuplumab (BMS-936564), 0.1% of H_2_O_2_ and 10 μM Fludarabine (F-ara-A) with 30 mM of Tiron for 4 hrs at 37°C. To measure ROS production, following CD19/CD5 labeling, cells were labeled with hydroxyethidium dye and incubated for 20 minutes at room temperature followed by flow cytometry analysis. The data presented represent mean ROS production± S.D. **C**. The cells were incubated alone with Tiron (30 mM) or 10 μg/ml of either rituximab, obinutuzumab, Ulocuplumab (BMS-936564), 0.1% of H_2_O_2_ and 10 μM Fludarabine (F-ara-A) with 30 mM of Tiron for 4 hrs at 37°C. TheThe percentage (%) cell death was measured using CD19/CD5/Annexin-V labeling followed by flow cytometry analysis. The representative data for ROS and % cell death has been shown from the same patient. The data presented represent mean ROS production± S.D.

Because the mechanism of action related to the pro-apoptotic activity of Ulocuplumab (BMS-936564) appears to be caspase independent, we performed experiments to address other possible explanations including induction of reactive oxygen species (ROS), which has been associated with monoclonal antibody induction of cell death [23]. CLL cells were incubated with Ulocuplumab (BMS-936564) or with controls (H_2_O_2_, Obinituzumab, F-ara-A, and Rituximab), and then were evaluated for superoxide production by using hydroxyethidium (HE) in conjunction with Annexin V to measure apoptosis. We observed that after 4 hrs of incubation, cells treated with Ulocuplumab (BMS-936564) showed a rapid increase in cell death and ROS production with levels that were significantly higher compared to untreated controls (*p* < 0.05). Positive controls for this experiment including H_2_O_2_ and Obinituzumab, an anti CD20 antibody known to induce ROS+ mediated apoptosis, showed an expected increase in ROS levels compared with untreated samples while isotype antibody control, rituximab and F-ara-A did not show an increase in ROS^+^/Annexin-V^+^ cells (Figure 7A). More over, ROS induction was a critical step required for Ulocuplumab (BMS-936564) to induce apoptosis. This was demonstrated when Tiron, a ROS inhibitor that has been extensively used in the previous studies [23-25], completely abrogated the ROS production and apoptosis induced by Ulocuplumab (BMS-936564) in leukemia cells (Figure 7B and 7C).

## DISCUSSIONS

Chemokines and chemokine receptors are important for the migration of immune cells to the site of inflammation and play an important role maintaining the homeostasis in the tumor microenvironment [26, 27]. Particularly, CXCR4 expression in CLL regulates cell migration, homing to lymphoid tissues and could favor the formation of minimal residual disease after treatment by inducing a “protective” anti-apoptotic microenvironment [28]. Therefore, the cellular signaling mediated by CXCR4 constitutes a potential biological target in CLL and other malignancies.

Ulocuplumab (BMS-936564) is a first in class, fully human IgG4 monoclonal antibody that has been engineered to specifically bind to CXCR4 [17]. *In vitro* studies have shown that Ulocuplumab (BMS-936564) has a potent anti-tumor activity in established tumors including AML, NHL, and multiple myeloma xenograft models [17]. In addition, preliminary reports from clinical studies in hematological malignancies using this antibody have shown encouraging clinical activity [29]. However, because the precise mechanism(s) of action of this antibody is not completely understood, we focused our studies to provide additional insights regarding this particular question.

We found that primary leukemia cells from CLL patients have significantly higher levels of CXCR4 expression compared with normal B cells and that high-risk prognostic factors do not influence the level of expression of this chemokine receptor. None of the patients were treated in the phase-I clinical trial for Ulocuplumab (BMS-936564). This is in agreement with previous reports from our group and others [30-32]. More over, CLL cells did not show expression of CXCL12 (CXCR4 ligand) by flow cytometry and *CXCL12* gene expression by RT-PCR was also negative in the large majority of samples. This suggests that activation of CXCR4 induced by CXCL12 occurs either by cell-cell interactions with non-leukemia CXCL12 expressing cells, likely located in lymphatic tissues, or mediated by soluble CXCL12.

Ulocuplumab (BMS-936564) showed a nanomolar binding affinity to CXCR4 expressed in Ramos cells with a CXCR4 receptor concentration that was 3-4 times higher than peripheral blood mononuclear cells (150,000 vs. 61,000-receptor density / cell). The pro-apoptotic activity of Ulocuplumab (BMS-936564) was dependent on membrane expression of CXCR4, and as expected for an IgG4 antibody, this molecule did not induce ADCC or CDC effector functions in Ramos or CLL cells.

Ulocuplumab (BMS-936564) induced CLL death regardless of the presence of prognostic factors that we used to segregate CLL-HR and CLL-LR [ZAP-70 expression, IgVH gene mutation status, *TP53mut* /Del(17p)] patients. However, in this cohorts of patients analyzed, we noticed that patients with *TP53mut* /Del(17p) have a lower response compared to other CLL samples. Despite of that, cell death induced by Ulocuplumab (BMS-936564) in this group of high-risk patients was significantly better than the low response observed with F-ara-A (*p < 0.0001*). These results suggest that the mechanism of cell death associated with Ulocuplumab (BMS-936564) is, at least in part, p53 independent, a finding that have significant clinical implications particularly in the treatment of refractory cancer where the majority of cases are associated with p53 dysfunction [33]. Among other anti-CXCR4 antibodies tested, 12G5 and 1D9, only 12G5 was able to induce cell death in CLL alone or co-cultured with stroma-NK-tert cells support, but the effect was weaker as compared to Ulocuplumab (BMS-936564). The reason for poor cytotoxicity of 1D9 may be because unlike 12G5, it does not compete for CXCL12 binding to CXCR4 [19, 34, 35].

Ulocuplumab (BMS-936564) binds to CXCR4 and blocks CXCL12 induced cytoskeletal changes and migration similarly to AMD3100. However, the lack of direct anti-cancer activity shown by AMD3100 and other synthetic peptide CXCR4 inhibitors, [6, 31, 36] suggest that binding to CXCR4 and inhibition of CXCR4-CXCL12 signaling is not sufficient to trigger cell death. This is further supported by evidence that Ulocuplumab (BMS-936564) induced similar levels of cell death in CLL cells cultured alone (lacking CXCL12 stimulation) or co-cultured with CXCL12 expressing stromal cell support.

We observed that Ulocuplumab (BMS-936564) induces pancaspase activation (caspases 2, 3, 8, 9) but this is not sufficient to induce cell death. This is supported by the fact that the potent caspase inhibitor Z-VAD did not block cell death induced by this antibody as it did with chemotherapy treated controls (etoposide and F-ara-A).

Caspase-independent cell death has been recognized as an alternative pathway that involves proteins released as a result of mitochondrial outer membrane permeabilization (MOMP) [37, 38]. These proteins including AIF, HtrA2/Omi, and endonuclease G, can undergo nuclear translocation to induce an early chromatic condensation, ROS production, DNA damage, lysosome activation and proteolysis mediated by release of cathepsin and proteases.

Because our data suggested the presence of this caspase-independent mechanism, we performed experiments to analyze the role of ROS in Ulocuplumab (BMS-936564) mediated cell death. We found that upon incubation with Ulocuplumab (BMS-936564), CLL cells generated ROS that were detected using dihydroethidium (HE) staining. HE is a cell-permeable fluorogenic probe that reacts with ROS to form ethidium, which intercalates within double-stranded DNA in the nucleus and emits red fluorescence. Cells that release ROS also underwent cell membrane changes associated with apoptosis including phosphatidylserine (PS) translocation from the inner to the outer leaflet of the cellular membrane that was detected by fluorescent staining with annexin V. The pattern of ROS production and apoptosis induced by Ulocuplumab (BMS-936564) was similar to obitunutuzumab (Gazyva), another antibody that has been recently shown to induce ROS dependent cell death. More over, Tiron, a well-known peroxide inhibitor/scavenger, was capable of rescuing cells from Ulocuplumab (BMS-936564) induced cell death confirming that ROS production is essential for this antibody induction of apoptosis.

Overall, we show here that Ulocuplumab (BMS-936564), a specific anti-CXCR4 antibody, binds to CXCR4 with low affinity and blocks CXCL12 mediated activation-chemotaxis. This antibody induces apoptosis in CLL cells regardless of the presence of high-risk prognostic factors in a p53 independent manner. In addition, Ulocuplumab (BMS-936564) induces cell death by a caspase-independent mechanism that involves ROS release. These data highlight the relevance of the CXCR4-CXCL12 pathway as a target in cancer therapy and provide additional rationale for ongoing clinical studies using Ulocuplumab (BMS-936564) in hematological malignancies including CLL.

## MATERIALS AND METHODS

### Collection and isolation of PBMCs from CLL patients

Peripheral_3_ blood mononuclear cells (PBMC) from CLL patients were obtained from the CLL Research Consortium (CRC) tissue repository, Moores Cancer center, University of California, San Diego (UCSD), La Jolla. After CLL diagnosis was confirmed, [39] patients provided written informed consent for blood sample collection on a protocol approved by the Institutional Review Board of the Moores UCSD Cancer Center, in accordance with the Declaration of Helsinki [40].

The details of the cell culture for primary CLL and different cell lines (Table 1), flow cytometry based CXCR4, CXCL12 profiling, cell death, apoptosis measurement, CXCR4 binding, F-actin polymerization, migration, and ROS detention and inhibition, Z-VAD assay for caspase activation, [41] and dependency assays are provided in the, “Supplementary Materials and Methods”[42-44]. Leukemia samples from patients with CLL were divided in two subgroups *i.e.* high risk (CLL-HR) and low risk (CLL-LR), based on the presence of high-risk prognostic makers including ZAP-70, CD38, and IGHV mutational status.

#### Calculation of specific induced apoptosis (SIA)

In order to discriminate the compound specific induced apoptosis vs. background spontaneous cell death from *in vitro* culture conditions, we calculated the percentage of specific induced apoptosis (% SIA) using the following formula: % SIA = [(compound induced apoptosis - media only spontaneous apoptosis) / (100- media only spontaneous apoptosis)] × 100.

### Statistical analysis

The data sets were analyzed using GraphPad Prism software (v. 5.0c; San Diego, CA). The Statistical significance was determined by using paired or unpaired Student's t test or one-way ANOVA followed by Bonferroni correction's multiple comparisons test. Statistical differences for the mean values are indicated as follows: *, *p* < 0.05; **, *p* < 0.01; ***, *p* < 0.001; and ****, *p* < 0.0001. The IC_50_ value was defined as the drug concentration that inhibits 50% cell growth compared with untreated controls and calculated by Graphpad Prism 6.0 software. Unless indicated, data are presented as the mean ± SEM.

## SUPPLEMENTARY MATERIALS AND METHODS FIGURES


