# Supplementary material for: Ulocuplumab (BMS-936564 / MDX1338): a fully human anti-CXCR4 antibody induces cell death in chronic lymphocytic leukemia mediated through a reactive oxygen species-dependent pathway
Source: Oncotarget. 2015 Dec 4;7(3):2809–22. doi: 10.18632/oncotarget.6465 (PMC4823073; doi:10.18632/oncotarget.6465)
Supplement: Supplementary file 1 [file oncotarget-07-2809-s001.pdf]

Ulocuplumab (BMS-936564 / MDX1338): a fully human anti-CXCR4 antibody induces cell death in chronic lymphocytic leukemia mediated through a reactive oxygen species-dependent pathway

Supplementary Materials and Methods

Kashyap et al (2015). Ulocuplumab (BMS-936564 / MDX1338): A Fully Human Anti-CXCR4 Antibody Induces cell death in Chronic Lymphocytic Leukemia Mediated Through a Reactive Oxygen Species-Dependent Pathway

Cell Culture

Peripheral blood mononuclear cells (PBMC) from patients with CLL were obtained from the CLL Research Consortium tissue bank. After CLL diagnosis was confirmed,[1] patients provided written informed consent for blood sample collection. The institutional review board of the University of California, San Diego, approved the protocol. PBMCs were isolated by Ficoll-Hypaque gradient density centrifugation (Cat# 17-1440-03, GE Healthcare Life Science) and used fresh or viably frozen and stored in liquid nitrogen for later use. Normal peripheral blood samples were obtained from the San Diego blood bank and PBMCs isolated and stored in the same manner. The isolation of B cells was done by positive selection using Dynabeads CD19 pan B (Cat# 11143D, Invitrogen) and DETACHaBEAD CD19 (Cat# 12506D, Invitrogen) according to the manufacturer's protocol.

Cell line culture

For cell culture, leukemia cell lines Raji (Burkitt's lymphoma, *TP53* mutant), Ramos (Burkitt's lymphoma, mutant type *TP53*), and Jurkat (T-Cell leukemia, mutant *TP53*) were obtained from ATCC and maintained in RPMI supplemented with 10% fetal bovine serum (FBS), 2 mM L-glutamine, and 100 U/mL of penicillin and 100 µg/mL of streptomycin at 37°C in an atmosphere of 5% CO_2. Different cell lines and their culture conditions are mentioned in ***Supplementary Table 1.***

Chronic Lymphocytic Leukemia (CLL) sampling and cell culture

CLL cells and PBMCs were separated from heparinized venous blood by density gradient centrifugation using Ficoll-Hypaque media (GE Healthcare). Samples with > 95% double positive cells for CD5 and CD19, as assessed by flow cytometry, were selected and used.

CLL cells were maintained in RPMI 1640 medium supplemented with 10% FBS, 2 mM L-glutamine, and 100 U/mL of penicillin and 100 μg/mL of streptomycin at 37 °C in an atmosphere containing 5% CO_2.

Normal B cell isolation

Normal B cells were purified from buffy coats of healthy volunteer donors, obtained from San Diego blood bank. Positive isolation with Dynabeads CD19 pan B (Life Technologies) and DETACHaBEAD CD19 (Life Technologies) were used to achieve more than 95% purity by flow cytometry analysis.

Cell cultures and stromal cell co-culture conditions

Briefly, primary leukemia cells derived from CLL patients were cultured in RPMI supplemented with 10% heat inactivated FBS (fetal bovine serum, Catalog # FB-02, Omega Scientific, Tarzana, CA) and 1% antibiotic at a density of 3×10^5 cells per milliliter at 37°C and 5% CO_2. Primary CLL cells were cultured alone in 96-well round bottom plates (Catalog # 3596, Corning, NY) or co-cultured with stroma-NK-tert cells in 96-well flat bottom plates (Catalog # 3799, Corning, NY) with a ratio of 20:1 (stroma-NK-tert:CLL) by seeding 300,000 cells/well. [2] Stroma-NK-tert cells are fibroblast derived from human bone marrow and immortalized with human telomerase reverse transcriptase (hTERT) bearing exogene MFG-tsT-IRES-neo (RCB2350- RIKEN Bioresource Center, Japan).

Ramos, Raji, K562, and Jurkat cells were grown in RPMI with 10% FBS and 1% antibiotic. Co-cultures with stromal cells were performed as previously described.[3] The human mesenchymal NK-tert stromal cell line was derived from bone marrow. Cells derived from the stroma-NK-tert cell line were immortalized with human telomerase reverse transcriptase (hTERT)] containing exogene MFG-tsT-IRES-neo. These cells were obtained from the RIKEN Cell Bank (RIKEN, Yokohama, Japan).[4] Stroma-NK-tert cells and other adherent cell lines (293 and Hela) were grown in DMEM (Catalog # 10-017-CV, Corning Cellgrov, Manassas, VA 20109) supplemented with 10% FBS and 1% antibiotic.

CXCR4 phenotyping in primary CLL samples and cell lines

The phenotyping of CLL cells for CXCR4 expression was carried out by flow cytometry using a 1:200 dilution of PE mouse anti-human CD184 Antibody (Catalog # 555974, BD Biosciences). The isotypic control was PE mouse IgG2a, k isotype control (Catalog # 555574, BD Biosciences).

CXCL12 phenotyping in primary CLL samples and cell lines

CXCL12 does not harbor any transmembrane domains, but does contain a signal peptide motif that can be detected as a secreted protein. To evaluate CXCL12 expression we did intracellular staining using the Cytofix/Cytoperm kit (Catalog # 51-2090KZ, BD Biosciences, San Diego, CA). The samples were washed with BD Perm/Wash buffer (Catalog # 51-2091KZ, BD Biosciences, San Diego, CA) and incubated for 30 minutes at room temperature with a 1:100 dilution of anti-CXCL12-APC tagged antibody (Catalog #IC350A, R&D System). The samples were then washed twice with FACS buffer (phosphate buffered saline containing 0.5% BSA plus 0.1% NaN_3) and analyzed by flow cytometry. APC-mouse IgG1k (Catalog # 555751, BD Biosciences) was used as an isotype control with a dilution of 1:100.

CXCR4 binding assays

Iodination of Ulocuplumab (BMS-936564) for scatchard analysis

Ulocuplumab (BMS-936564) was radioiodinated using IODO-GEN® solid phase iodination reagent (1,3,4,6-tetrachloro-3a-6a-diphenylglycouril; Pierce). Excess iodide was removed using a desalting column. Fractions of labeled antibody were collected and analyzed for radioactivity on a Wizard 1470 gamma counter (PerkinElmer). Radiopurity was established by thin layer chromatography of peak protein and radioactive fractions (Catalog # 15-005, Pinestar Technology). The ^{125}I-Ulocuplumab (BMS-936564) concentrations from the most radiopure fractions were measured in the Qubit™ Fluorometer (Invitrogen) using the Quant-iT™ reagent. A standard curve of counts per minute (CPM) corresponding to the molar concentration of ^{125}I-Ulocuplumab (BMS-936564) was plotted.

Affinity and saturation binding of ^{125}I- Ulocuplumab (BMS-936564)

Target cells were washed and adjusted to 8 x 10^6 cells/mL in binding buffer (24 mM Tris pH 7.2, 137 mM NaCl, 2.7 mM KCl, 2 mM glucose, 1 mM $CaCl_2$, 1 mM $MgCl_2$, 0.1% BSA). Overnight buffer-coated (1% milk/PBS) glass fiber plates (Catalog # MAFBNOB50, Millipore) were washed 3 times with 0.2 mL of binding buffer. Fifty microliters of buffer alone was added to the maximum binding wells (total binding) and control wells. 25 µL of varying concentrations of ^{125}I-Ulocuplumab (BMS-936564) were added to all wells. 25 µL of varying concentrations of unlabeled antibody at 100-fold excess was added to control wells to determine nonspecific binding (NSB). 25 µL of cells suspended in binding buffer was added to all wells to bring the total volume to 50 µL. The plates were incubated for 2 hours at 4°C followed by three washes with 0.2 mL cold wash buffer (24 mM Tris pH 7.2, 500 mM NaCl, 2.7 mM KCl, 2 mM glucose, 1 mM $CaCl_2$, 1 mM $MgCl_2$, 0.1% BSA). Filters were removed and radioactivity was measured on a Wizard® gamma counter (PerkinElmer). The CPM generated by the competition with unlabeled Ulocuplumab (BMS-936564) was subtracted from the CPM generated by the un-competed ^{125}I-Ulocuplumab (BMS-936564) binding to yield specific binding. Saturation of ^{125}I-Ulocuplumab (BMS-936564) binding to human cell lines was confirmed based on the standard curve of ^{125}I-Ulocuplumab (BMS-936564) concentrations versus gamma CPM. Equilibrium binding (K_D value) was calculated from the specific binding curve using GraphPad Prism software with one-site binding parameters.

Antibody Dependent Cellular Cytotoxicity (ADCC) assay in CLL

The ADCC assay was performed as per the instructions of the manufacturer. We used the ADCC Reporter Bioassay kit from Promega (Catalog #G7010). Jurkat cells were provided in the promega kit. The transfected Jurkat cell line was grown in RPMI containing G-418 sulfate solution (Catalog # V8091) and hygromycin (Catalog # 10687010, 50 mg/ml solution). The ADCC buffer (99.5% RPMI 1640 with L-glutamine and 0.5% super low IgG FBS) was prepared using RPMI supplemented with Super low IgG defined fetal bovine serum (catalog # SH30898, Hyclone). The luciferase assay system was used as a read-out (Catalog # G7940, Promega). Different concentrations of isotype controls (IgG4 for BMS-

936564, and IgG1 for rituximab) and BMS-936564 (highest concentration at 10 μg/ml) were added to the effector/target cell mixtures in a ratio of 1:1. Different ratios were tested for Effector: target to minimize the background of effector (Jurkat cells express CXCR4), different ratios were tested and based on the background in response to BMS-936564 treatment, the 1:1 ratio of effector to target was chosen. First, the dilutions were made in a V-shaped 96-well plate (catalog # 3894, Costar). The effector cells were harvested (we always used fresh cells, not the frozen one) for all the ADCC assays. Cells were counted and then washed twice with PBS to get rid off of the high IgG FBS serum. We used a ratio of 1: 1 for effector: target cells. The number of cells used was 75,000 for either effector or target cells. A volume of 50 ul of the mixture of effector: target cells were plated in a 96 well flat plates with white color wells (Catalog # 3917, Corning Life Sciences) using a multi-channel pipette without creating bubbles. Finally, 25 μl of antibody, isotype, and ADCC buffer were added in the respective wells from the V-shaped 96-well plate and incubated for 6 hrs at 37°C in a humidified CO_2 incubator. Following incubation, the plate was equilibrated to ambient temperature for 15 minutes. 75 μl of Bio-GloTM luciferase assay reagent was added into each well without creating bubbles and incubated at room/ambient temperature for 30 minutes. The luminescence was detected using an Infinite 200 Microplate Reader (Tekan). Results are expressed in relative light units (RLU).

CDC assay on CLL cells

BMS-936564, obinutuzumab or Rituximab was added to CLL-B cells (1×10^{6} /mL) in complete medium supplemented by 5% human serum. The serum was incubated at 56°C for 30 minutes to inactivate the complement. The heat inactivated /normal human serum treated cells were incubated for 4 hrs at 37°C. The % SIA was determined using flow cytometry after PI/DiOC6 staining. % SIA calculated according to the following formula: 100 × (% viable cells with inactivated serum − % viable cells with native serum)/(% viable cells with inactivated serum), all measures being taken after the 4-hour incubation.

ADCC assay

PBMC effector cells were purified from heparinized whole blood by standard Ficoll-Paque separation and cultured overnight in the presence of 50 U/mL IL-2 (Catalog # 202-IL-050,

R&D Systems). Ramos cell line was used as a target cell line. The cells were labeled with 20 mM of bis(acetoxymethyl)2,2':6',2"-terpyridine-6,6"-dicarboxylate (BADTA, cat # C136-100, PerkinElmer) and added to effector cells for a final target: effector ratio of 1:50. Ten-fold serial dilutions of Ulocuplumab (BMS-936564), rituximab and isotype controls (beginning at 0.0001 µg/mL) were added to the effector/target cell mixtures. After 1 hour incubation at 37°C, 20 µL of supernatant was harvested from each well and mixed with 180 µL europium solution (Cat # C135-100, PerkinElmer) in a 96-well flat bottom plate. The reaction was read with a Fusion-Alpha TRF reader using a 400-microsecond delay with 330/80 excitation and 620/10 emission filters. Target cells without effector cells and no antibody provided the control for background spontaneous dye release while target cells plus effector cells lysed with methanol represented maximal release. Antibody-dependent percent specific lysis was calculated with reference to these controls and was plotted versus antibody concentration with a nonlinear regression analysis using GraphPad Prism software.

CDC assay

Ramos target cells (5×10^4 cells/mL) were mixed with a 1:3 dilution of human complement (Catalog # A113, Quidel) and plated 50 µL/well in a 96-well plate. Ulocuplumab (BMS-936564) and isotype control antibody were serially diluted 5-fold from 50 µg/mL to 3.2 ng/mL and dispensed into the cell/complement mixture in duplicate. Assay plates were incubated at 37°C for 2 hours before the addition of alamarBlue (Catalog # DAL1100, Biosource). Alamar Blue is a redox indicator that yields a colorimetric change and a fluorescent signal in response to metabolic activity to measure cell viability. The plates were incubated for an additional 21 hours at 37°C and read on a Spectra Max Gemini fluorescent plate reader (Molecular Devices, Sunnyvale, CA; EX530 EM590). Viable cell count was considered proportional to fluorescence units (FLU) and was plotted against antibody concentration with a nonlinear regression analysis using GraphPad Prism software.

Flow Cytometry

The flow cytometric data collection and analysis was carried out using FACScalibur (BD Biosciences, San Jose, CA) and FlowJo software (version 9, TreeStar Inc, Ashland, OR).

Detection of apoptosis

Apoptotic and viable cells were discriminated by *staining the* cells with a 1:1000 dilution of 40 uM 3,3'-dihexyloxacarbocyanine iodide (DiOC6) (Catalog #D-273, Molecular Probes, Eugene, OR, USA) and the same dilution of 1 mg/ml propidium iodide (Catalog # 81845, Sigma, St Louis, MO, USA) for 30 minutes at 37°C. Using this method, viable cells exclude PI and stain brightly positive for DiOC6.[5] CLL B cell specific gating was done using a 1:40 dilution of CD19-PerCP-Cy5.5 (catalog # 8045-0198, clone SJ25C1, eBioscience, San Diego) and a 1:50 dilution of APC mouse anti-human CD5 (Catalog # 555355, clone UCHT2, BD Biosciences) specific antibodies. The CLL cells were subsequently stained with DiOC6 at 37°C for 30 minutes and subjected to flow cytometry analysis. Normal B and T cells were gated using a 1:40 dilution of CD19-PerCP-Cy5.5 for B cells and a 1:50 dilution of PE mouse anti-human CD3 (Catalog # 555333, clone UCHT1, BD Biosciences) for T cells. The commercial anti-CXCR4 antibodies tested for in vitro cytotoxicity were 1D9 clone (rat IgG2a,κ, catalog # 551413, BD Biosciences), and 12G5 clone (monoclonal mouse IgG2A, catalog # MAB170, R&D Systems).

Inhibition of actin polymerization

The Ulocuplumab (BMS-936564) antibody was assessed for its ability to inhibit actin polymerization in primary leukemia cells from patients with CLL. Actin polymerization is a surrogate marker of cancer cell migration, metastatic potential and stromal cell dependency induced by the interaction of CXCR4 with CXCL12.[6] CLL cells were thawed and resuspended in RPMI-1640 medium with 0.5% BSA and allowed to rest for 1-2 hours 37°C. Cells were then incubated at 37°C with the Ulocuplumab (BMS-936564) or AMD3100 (Mozobil) for 1 hour. After treatment, each sample was stimulated with 90nM CXCL12 (SDF-1α, catalog # 300-28A, PeproTech, Rocky Hill, NJ) at 37°C for 15 seconds. After stimulation, a solution containing $4x10^{-6}$ M FITC-labeled Phalloidin from Amanita phalloides (Catalog # P5282, Sigma Chemical, St. Louis, MO), 0.5 mg/mL 1-α-lysophosphatidylcholine (Catalog # L4129, Sigma, St. Louis, MO), and 10% formaldehyde

(methanol free; Catalog # 04018, Polysciences, Inc., Warrington, PA) in phosphate-buffered saline (PBS) was added. The fixed cells were analyzed by flow cytometry on a FACSCalibur within one hour. The data was expressed as the mean relative intensity fluorescence (MFI) of the sample after addition of CXCL12 divided by the MFI of an identical sample with no CXCL12 stimulation. The anti-CXCR4 antibody was evaluated for its ability to inhibit actin polymerization induced by CXCL12.

Inhibition of migration of cells in a transwell assay

Ulocuplumab (BMS-936564) was assessed for its ability to inhibit CXCL12 induced chemotaxis in primary leukemia cells from CLL patients using a transwell migration assay. CLL cells were thawed and resuspended in RPMI-1640 medium supplemented with 10% FBS and 0.5% BSA, and allowed to rest overnight at 37°C. Cells were then incubated at 37°C with Ulocuplumab (BMS-936564) or AMD3100 (Mozobil) for 1 hour. 5 x 10^5 pre-treated CLL cells in a 100 μl volume were then loaded into the top chamber of a 5.0 μm polycarbonate membrane transwell permeable support (Catalog # 978-17893, Costar, Corning, NY) and 600 μl of 12.5 nM (100 ng/ml) CXCL12 (SDF-1α, catalog # 300-28A, PeproTech, Rocky Hill, NJ) was added to the lower chamber. A 1:20 dilution of the pre-treated cell mixture loaded into the top chamber was set aside to count by FACS. The transwell plate was then allowed to incubate at 37°C for 2 hours, followed by removing 200uL from the lower chamber and counting the number of migrated cells by flow cytometry using a FACSCalibur. Cell counts were determined by the number of events recorded over a 20 second period using the MED flow rate (35 μl/min). The flow rate and cell counts were then used to extrapolate how many cells were initially loaded into the top chamber (using the 1:20 dilutions saved previously) as well as how many cells had migrated to the lower chamber after the 2 hours incubation, allowing us to determine the percentage of cells that migrated.

Caspase dependent / independent Mechanism of Cell Death by Caspase Colorimetric Protease Assay

For examination of mechanism of cell death of Ulocuplumab (BMS-936564), treated CLL cells were screened for different caspase activation including caspase-2, caspase-3, caspase-6, caspase-8, and caspase-9 using the ApoTarget Caspase Colorimetric Protease

Assay Sampler kit (Cat # KHZ1001, Invitrogen, Frederick, MD) according to the manufacturer's instructions. CLL cells (5.0×10^6) were treated with or without the Ulocuplumab (BMS-936564) – 200 nM, isotype control (200 nM), and a control cell death inducing agent (e.g. Etoposide and Fludarabine) for 6 hrs at 37°C. Following treatment, cells were washed twice with chilled PBS and resuspended in 50 μl of ice-cold cell lysis buffer for 10 minutes on ice. The lysates were centrifuged at 10,000 rpm for five minutes to remove cell debris. The protein concentration for each sample was measured using NanoDrop equipment (*NanoDrop ND-1000,* Wilmington, DE, USA). For the caspase detection assay, 100 μg of protein from each sample was added to a 96-well flat bottom ELISA microplate. 50 μl of reaction buffer and 5 μl of each caspase-substrate [200 μM VDVAD-*p*NA (caspase-2), DEVD-*p*NA (caspase-3), VEID-pNA (Caspase 6), IETD-*p*NA (caspase-8), and LEHD-*p*NA (caspase-9)] was added followed by incubation at 37°C for 2 hours. Following the cleavage of the substrates by different caspases, absorption for the free pNA was quantified using an ELISA reader (Catalog # 432789,VERSAmax model, Molecular Devices, Sunnyvale, California) at 405 nm. Comparisons of the absorbance of pNA obtained from treated and untreated samples allowed determination of the fold change in the caspase activity. The data was derived from two independent experiments including technical replicates.

Detection of reactive oxygen species (ROS) by flow cytometry

CLL cells were seeded at $2.5\text{x}10^5$/ml in RPMI media and treated with 10 μg/ml of Ulocuplumab (BMS-936564) for 4 hours at 37°C and 5% CO_2 in 24-well plates. The generation of ROS was detected using dihydroethidium (HE) staining (Catalog # D1168, Sigma-Aldrich, St. Louis, MO). HE is a cell-permeable fluorogenic probe that reacts with ROS to form ethidium, which intercalates within double-stranded DNA in the nucleus and emits red fluorescence. Cells were re-suspended in phosphate buffered saline (PBS) containing 2.5 μM of HE dye and incubated for 30 minutes in the dark at room temperature. Cells were then washed with annexin V binding buffer (AVB) (catalog # 51-66121E, BD Bioscience) twice and stained with a 1:100 dilution of annexin V (catalog # 556421, BD Biosciences) in AVB at room temperature for 15 minutes. The samples were then analyzed by flow cytometry followed by data analysis using FlowJo software. Further, to test

whether ROS specificity, we used ROS inhibitor Tiron (Catalog # 172553, Sigma-Aldrich) at 30 mM concentration. The CLL cells were incubated with BMS-36564, rituximab, GA101, and F-ara-A in absence or presence of Tiron for 6 hrs. The CLL cells were assessed for ROS production by CD19/CD5/HE staining and cell death by CD19/CD5/Annexin-V staining followed by flow cytometry analysis.

References

1. Matutes E, Owusu-Ankomah K, Morilla R, Garcia Marco J, Houlihan A, Que TH*, et al.* The immunological profile of B-cell disorders and proposal of a scoring system for the diagnosis of CLL. *Leukemia* 1994 Oct; **8**(10)**:** 1640-1645.

2. Tsukada N, Burger JA, Zvaifler NJ, Kipps TJ. Distinctive features of "nurselike" cells that differentiate in the context of chronic lymphocytic leukemia. *Blood* 2002 Feb 1; **99**(3)**:** 1030-1037.

3. Kurtova AV, Balakrishnan K, Chen R, Ding W, Schnabl S, Quiroga MP*, et al.* Diverse marrow stromal cells protect CLL cells from spontaneous and drug-induced apoptosis: development of a reliable and reproducible system to assess stromal cell adhesion-mediated drug resistance. *Blood* 2009 Nov 12; **114**(20)**:** 4441-4450.

4. Kawano Y, Kobune M, Yamaguchi M, Nakamura K, Ito Y, Sasaki K*, et al.* Ex vivo expansion of human umbilical cord hematopoietic progenitor cells using a coculture system with human telomerase catalytic subunit (hTERT)-transfected human stromal cells. *Blood* 2003 Jan 15; **101**(2)**:** 532-540.

5. Ozgen U, Savasan S, Buck S, Ravindranath Y. Comparison of DiOC(6)(3) uptake and annexin V labeling for quantification of apoptosis in leukemia cells and non-malignant T lymphocytes from children. *Cytometry* 2000 Feb 15; **42**(1)**:** 74-78.

6. Burger JA, Kipps TJ. CXCR4: a key receptor in the crosstalk between tumor cells and their microenvironment. *Blood* 2006 Mar 1; **107**(5)**:** 1761-1767.

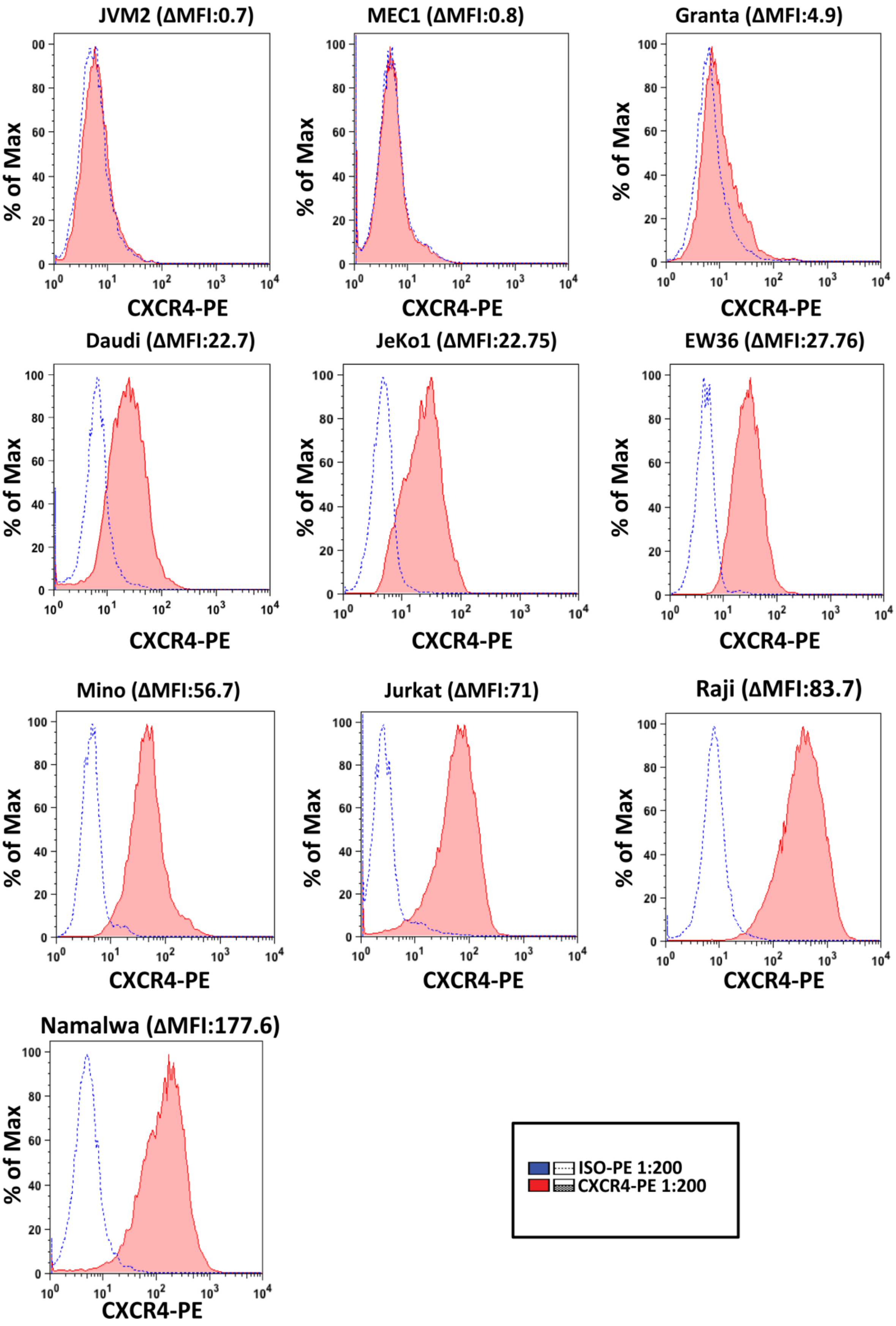

JVM2 (ΔMFI:0.7)
MEC1 (ΔMFI:0.8)
Granta (ΔMFI:4.9)
Daudi (ΔMFI:22.7)
JeKo1 (ΔMFI:22.75)
EW36 (ΔMFI:27.76)
Mino (ΔMFI:56.7)
Jurkat (ΔMFI:71)
Raji (ΔMFI:83.7)
Namalwa (ΔMFI:177.6)
% of Max
CXCR4-PE
ISO-PE 1:200
CXCR4-PE 1:200

Supplementary Figure 1: CXCR4 expression profiling in different cell lines

CXCR4 Expression profiling was done using an anti-CXCR4 antibody for surface staining in MEC1, K562, JVM2, Raji, Ramos, Jurkat, Granta, Daudi, Mino, Namalwa, and JeKo-1 cell lines followed by analysis of samples using flow cytometry. The CXCR4 expression is presented in form of ΔMFI.

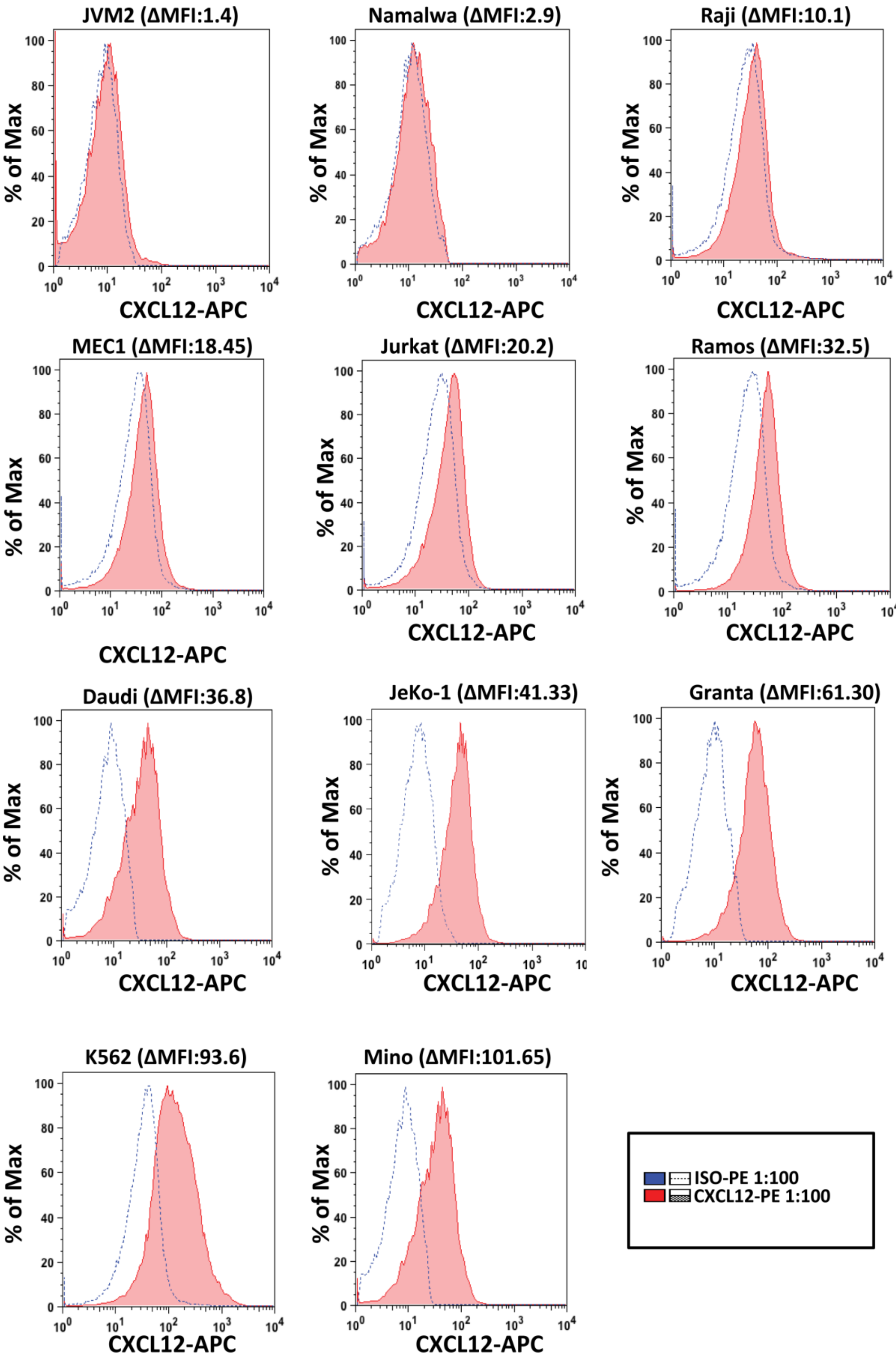
JVM2 (ΔMFI:1.4)
Namalwa (ΔMFI:2.9)
Raji (ΔMFI:10.1)
MEC1 (ΔMFI:18.45)
Jurkat (ΔMFI:20.2)
Ramos (ΔMFI:32.5)
Daudi (ΔMFI:36.8)
JeKo-1 (ΔMFI:41.33)
Granta (ΔMFI:61.30)
K562 (ΔMFI:93.6)
Mino (ΔMFI:101.65)
% of Max
CXCL12-APC
ISO-PE 1:100
CXCL12-PE 1:100

Supplementary Figure 2 : CXCL12 expression profiling in different cell lines.

CXCL12 Expression profiling was done using an anti-CXCL12 antibody for intracellular staining MEC1, K562, JVM2, Raji, Ramos, Jurkat, Granta, Daudi, Mino, Namalwa, and JeKo-1 cell lines followed by analysis of samples using flow cytometry. The CXCL12 expression is presented in form of ΔMFI. Intracellular expression of CXCL12 in JVM2, Namalwa, Raji, MEC1, Jurkat, Ramos, Daudi, JeKo-1, Granta, K562, and Mino (arranged in increasing order for CXCL12 expression) was covering a wide range of ΔMFI from 1.4 to 101.65.

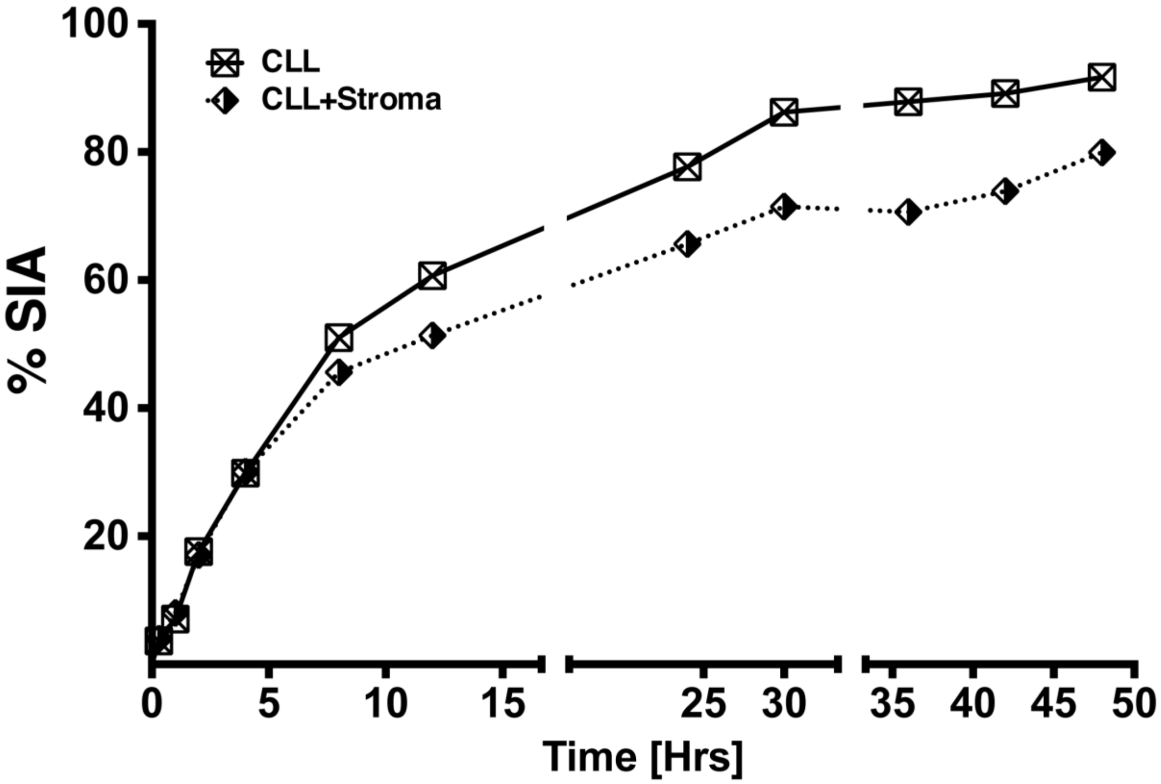

Supplementary Figure 3 : Washout experiment using Ulocuplumab (BMS-936564) in CLL alone or co-culture with stroma-NK-tert cells

In the washout experiment, CLL cells alone or co-cultured with stroma-NK-tert cells were incubated with Ulocuplumab (BMS-936564) at different time points. The cells were pelleted using centrifugation at 1100 rpm for 5 minutes, washed twice with RPMI (with 10% FBS) and equivalent volume of RPMI was added. The cells were allowed to go for incubation for 48 hrs. All incubations were performed at 37 °C. The cell death was measured in all the samples after 48 hrs using CD19/CD5/DioC6 staining followed by flow cytometry data analysis.

A.

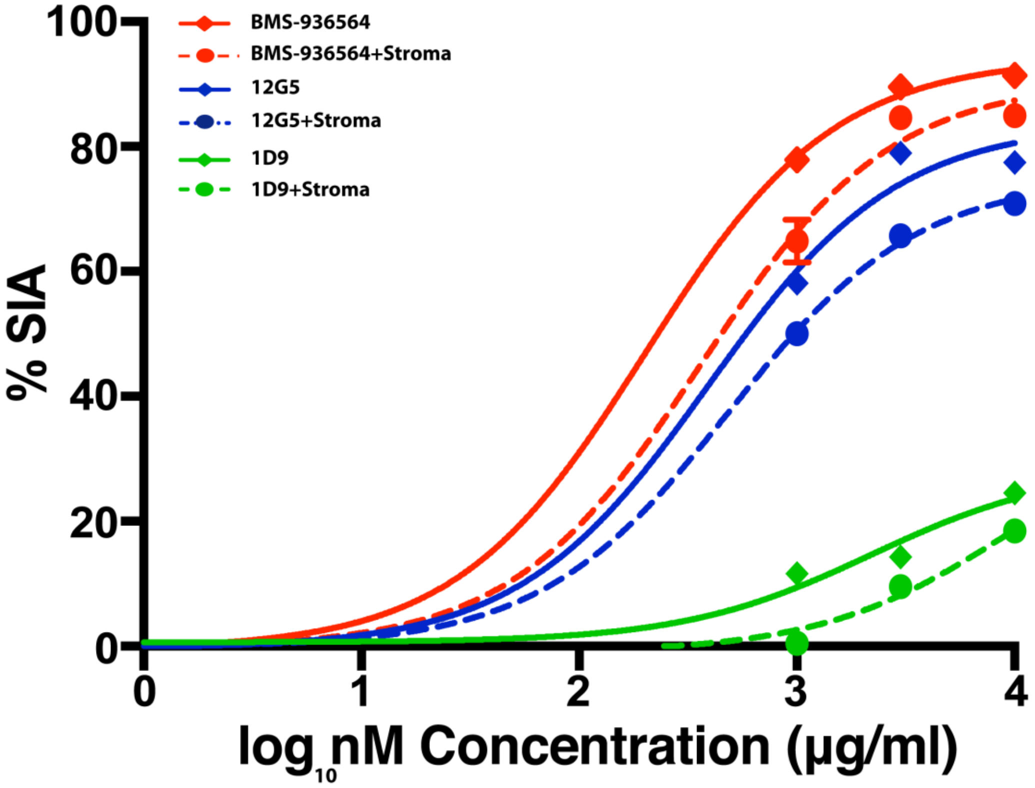

B.

IC_{50} (nM)	1D9	12G5	BMS-936564
**CLL**	Not achievable	32.135	12.43
**CLL+Stroma**	Not achievable	692.29	26.45
**1D9: rat IgG2a,κ; 12G5: mouse IgG2a; BMS-936564: human IgG4**			

Supplementary Figure 4: *In vitro* activity and IC_{50} of different anti-CXCR4 antibodies in CLL cells

Panel A. CLL B cells either alone or co-cultured with stromal cell support were incubated for 48 hrs with different anti-CXCR4 antibodies *i.e.* 1D9, 12G5, and Ulocuplumab (BMS-936564) with 1, 3, and 10 µg/ml of concentration. Following incubation, cells were labeled with CD19/CD5/Annexin V staining followed by flow cytometric analysis for *in vitro*

cytotoxicity. The cell death was presented in % SIA. **Panel B.** The IC_{50} for 1D9, 12G5, and Ulocuplumab (BMS-936564) antibodies has been mentioned in the table either after alone CLL or co-culture condition with stromal cell support.
